# A modeling method for the development of a bioprocess to optimally produce *umqombothi* (a South African traditional beer)

**DOI:** 10.1038/s41598-021-00097-w

**Published:** 2021-10-18

**Authors:** Edwin Hlangwani, Wesley Doorsamy, Janet Adeyinka Adebiyi, Lanrewaju Ibrahim Fajimi, Oluwafemi Ayodeji Adebo

**Affiliations:** 1 Department of Biotechnology and Food Technology, Faculty of Science, University of Johannesburg, Doornfontein Campus, P. O. Box 17011, Gauteng, South Africa; 2 Institute for Intelligent Systems, University of Johannesburg, Doornfontein Campus, P.O Box 17011, Gauteng, 2028 South Africa; 3 Department of Chemical Engineering, Faculty of Engineering and the Built Environment, University of Johannesburg, Doornfontein Campus, P.O. Box 17011, Gauteng, 2028 South Africa

**Keywords:** Biological techniques, Computational biology and bioinformatics, Developmental biology

## Abstract

Bioprocess development for *umqombothi* (a South African traditional beer) as with other traditional beer products can be complex. As a result, beverage bioprocess development is shifting towards new systematic protocols of experimentation. Traditional optimization methods such as response surface methodology (RSM) require further comparison with a relevant machine learning system. Artificial neural network (ANN) is an effective non-linear multivariate tool in bioprocessing, with enormous generalization, prediction, and validation capabilities. ANN bioprocess development and optimization of *umqombothi* were done using RSM and ANN. The optimum condition values were 1.1 h, 29.3 °C, and 25.9 h for cooking time, fermentation temperature, and fermentation time, respectively. RSM was an effective tool for the optimization of *umqombothi*’s bioprocessing parameters shown by the coefficient of determination (R^2^) closer to 1. RSM significant parameters: alcohol content, total soluble solids (TSS), and pH had R^2^ values of 0.94, 0.93, and 0.99 respectively while the constructed ANN significant parameters: alcohol content, TSS, and viscosity had R^2^ values of 0.96, 0.96, and 0.92 respectively. The correlation between experimental and predicted values suggested that both RSM and ANN were suitable bioprocess development and optimization tools.

## Introduction

The heterogeneous nature of food complicates food bioprocessing operations through varying responses to process conditions^1^. Thus, the development and application of dynamic optimization approaches is an important step towards ensuring robust process control, quality, and consumer safety^2,3^. The technical application of these approaches, especially in a biologically complex product such as traditional beer has been minimal^4^. Furthermore, variable microbial growth kinetics, process constraints, biochemical reactions, dynamic food matrices, and difficult bioprocessing requirements amplify complexities in bioprocess development and optimization^3,5^. As a result, the combination of linear and non-linear techniques is an effective approach to describe, analyze, and predict bioprocess responses that impact the outcomes of the final product^3,6^.

The use of a single technique may not be adequate in ascertaining the relationship between process input variables and the quality of the product^5^. Nonetheless, standalone mathematical and statistical models have been previously successful in describing the linear, interactive, and quadratic effects of selected parameters in beer bioprocessing^6,7^. Response surface methodology (RSM) and factorial experiment with their associated designs are traditional statistical models which have been applied extensively to screen and optimize factors in the biotechnology and food engineering industries^3,6^. RSM consists of a group of empirical techniques which evaluate the relationship between a group of control experiment parameters to achieve an optimal process^8,9^. In particular, RSM has been used as a statistical method to generate efficient models to optimize very large and complex bioprocesses in food systems^1,10^. RSM determines the significance of a model and defines the relationship between process variables through analysis of variance (ANOVA) and the lack of fit^1,11^. Moreover, the optimum conditions are determined by the desirability function^10,11^. However, traditional techniques show significant limitations in biological processes^3,6^. For example, RSM disregards parameters deemed insignificant without accounting for the possible interactive effects on the output of bioprocess.

Artificial intelligence (AI) and machine learning (ML) tools such as fuzzy logic, ANN, particle swarm optimization (PSO), and genetic algorithm (GA) are emerging technologies appropriate for the research and development of efficient bioprocesses^6,12^. Recently, the application of 2-step or 3-step optimization approaches involving RSM, ANN, and GA has become standard practice for manufacturing and other biological processes^7,12^. However, these tools and approaches have been rarely applied in the modeling and optimization of brewing and fermentation processes^3^. ANN has been successful in accurately approximating linear and non-linear functions from historic data devoid of cellular kinetics and metabolic fluxes, especially in multivariate bioprocesses^3,6^. ANNs are mathematical emulations of the biological learning process occurring within the brain. It can arithmetically model the network structure of interconnected nerve cells, and thus “learns”, link associations, and adapts to make accurate value predictions from a specific sample set^13^.

ANN possesses extraordinary processing abilities such as self-organization and data classification, pattern recognition, processing fuzzy and inaccurate information, good generalization capabilities, quicker processing time, noise and fault tolerance as well as high parallelism^12,13^. Given its numerous benefits, the use of ANN as a non-linear multivariate tool in bioprocess development can improve both the bioprocess and the final product^14,15^. For *umqombothi* bioprocessing, ANN presents a unique advantage in the improving developed RSM models since its standard framework has an inherent ability to use background information to solve problems^4,16^. Bioprocessing approaches that apply both RSM with adaptive learning techniques such as ANN have been shown to have better accuracy, prediction, and dependence relation when compared to traditional, isolated RSM^17,18^. As such, bioprocess development and optimization without carefully deliberated process designs will result in irreproducible and unreliable process designs^4,10^. In this study, a modeling method for the development of a bioprocess to optimally produce *umqombothi* was investigated.

## Methodology

### Traditional beer (***umqombothi***) brewing process

Five hundred (500) g of pre-packaged King Korn malted sorghum (*Mtombo – Mmela*) (Tiger Brands, Bryanston, South Africa) was mixed with 1000 g of White Star maize meal (Pioneer Foods, Bryanston, South Africa) in a sterile 10 L bucket filled with 7 L tap water. The mixture was gently stirred, covered, and incubated (Labcon, Chamdor, South Africa) at 25 °C for 24 h to sour. Thereafter, the soured paste was stirred gently and cooked for 30 min at 45 °C to make a traditional beer porridge (*isdudu*). The porridge was allowed to cool to 25 °C after which 500 g of King Korn malted sorghum (Tiger Brands, Bryanston, South Africa) was added and gently stirred. The mixture was then incubated at 30 °C (Labcon, Chamdor, South Africa) for 24 h to ferment. The finished beer was then tested for physicochemical properties.

### Experimental design using response surface methodology (RSM)

**Figure 1:**
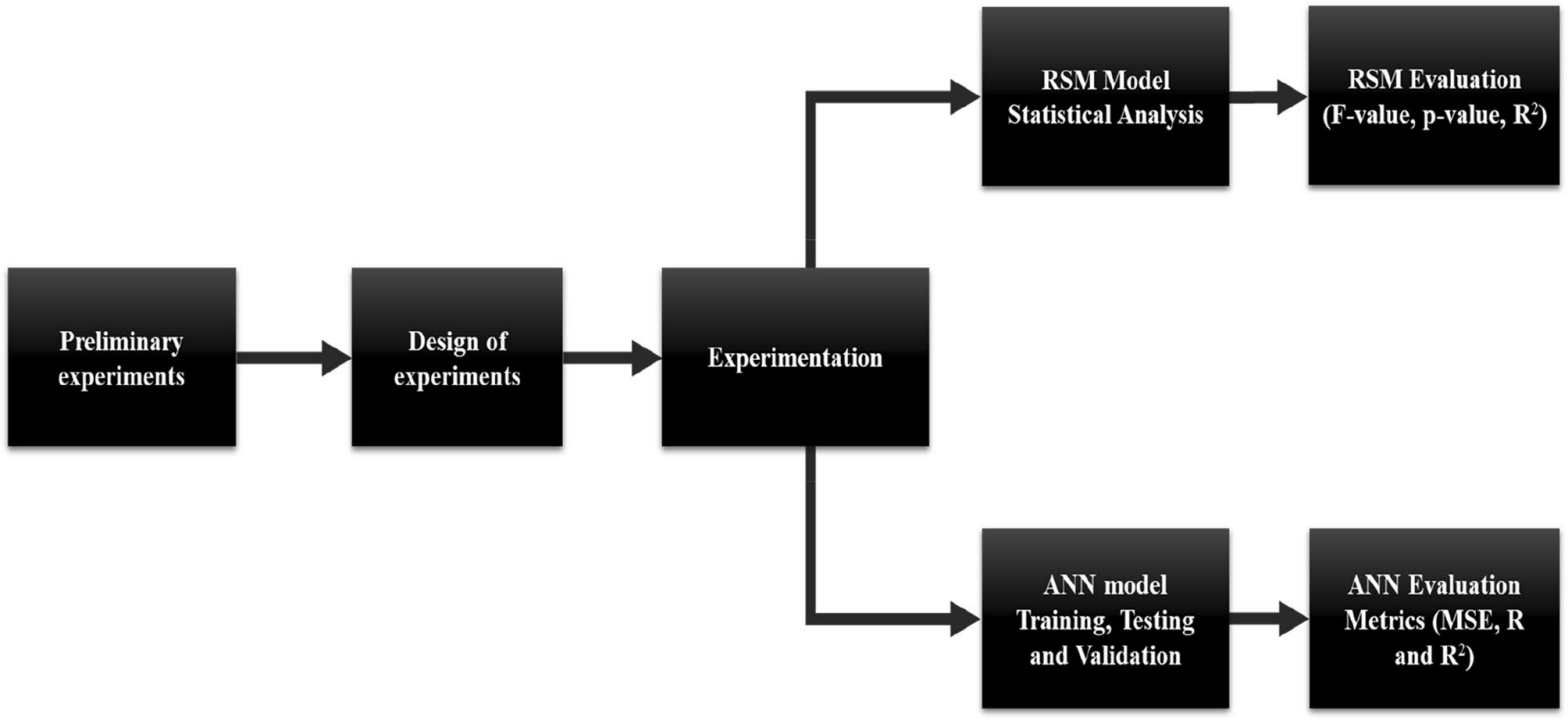
A flow chart of the complete experimental design and optimization techniques.

Preliminary experiments (data not presented herein) were conducted to determine appropriate ranges for processing factors: cooking time, fermentation temperature, and fermentation time and their effects on alcohol content, total soluble solids (TSS), total titratable acidity (TTA), pH, and viscosity in *umqombothi.* The obtained data was then used for the design of experiments (DOE) (Fig. 1). Thereafter, appropriate ranges were determined for factors of interest (Table 1). Central Composite Design (CCD) in Design-Expert software version 11.0.0 (Stat-Ease Inc., Minneapolis, USA) was used to generate 20 experimental runs. The input factors were cooking time (hr), fermentation temperature (°C), and fermentation time (hr) (Table 1). Following experimental combinations (Table 2) subsequent experiments were conducted.

**Table 1:** Process parameters selected for optimization: cooking time, fermentation temperature, and fermentation time.

Parameters	Code	High level (+ 1)	Medium level (0)	Low level (–1)
Cooking time (hr)	X_1_	3	2	1
Fermentation temperature (°C)	X_2_	35	30	25
Fermentation time (hr)	X_3_	96	60	24

*hr* hour.

**Table 2:** Experimental design of *umqombothi*.

Experimental run	Cooking time (hr)	Fermentation temperature (°C)	Fermentation time (hr)
1	2	38.41	60
2	2	30	60
3	3	35	24
4	2	30	60
5	2	30	60
6	3	25	24
7	2	30	60
8	1	35	96
9	3	25	96
10	1	25	24
11	2	30	60
12	1	25	96
13	3	35	96
14	3.68	30	60
15	2	21.59	60
16	1	35	24
17	0.32	30	60
18	2	30	120.54
19	2	30	60
20	2	30	0

*hr* hour.

Samples were withdrawn after each experimental run (done in triplicates) and alcohol content (°P), TSS (g/100 g), TTA (% lactic acid), pH, viscosity (cm/min) were determined. The Design-Expert software was also used to analyze and compute a second-order polynomial model to estimate and predict response values over a range of input parameter values by determining which input factors influenced responses, and the direction of that drive for the designed experiments as depicted in Eq. (1) below:

1 $$Y={\beta }_{o}+\sum {\beta }_{\dot{i}}{x}_{i}+\sum {\beta }_{ii}{x}^{2}+\sum {\beta }_{ij}{x}_{i}{x}_{j}+\varepsilon$$

where $Y$ indicated the response variable (optimal production parameter), ${\beta }_{o}$ the intercept of the response variable, while ${\beta }_{i}$, ${\beta }_{ii}$, and ${\beta }_{ij}$ were coefficients corresponding to the factor ${x}_{i}$, ${x}_{j}$ ($i,j$ = 1, 2, …, *n*). The input variables that affected the response $Y$ were ${\mathrm{x}}_{1}$,${\mathrm{x}}_{2}$, ${\mathrm{x}}_{3}$. The random error was represented by $\varepsilon$.

### Neural network construction and fitting

Experimental data was organized and used for the development of ANN prediction models. A matrix laboratory MATLAB R2020a (MathWorks, Massachusetts, USA) software was used for the design of function fitting neural network. A feed-forward neural network with two layers was used. The first layer was the input layer and the second layer was the output layer, both of which were triggered using the sigmoid activation function. Cooking time (hr), fermentation temperature (°C) and fermentation time (hr) were used as network inputs and alcohol content (°P), TSS (g/100 g), TTA (% lactic acid), pH, and viscosity (cm/min), were each used as the outputs to develop several networks and to determine the optimal network topology. Experimental data were randomly divided for training, validation, and testing. For training, 14 (70%) instances were used, 3 (15%) for validation and 3 (15%) for testing. The ANN model was then trained, validated, and tested by the Levenberg–Marquardt (LM) training algorithm. To further study the responses of the model, Bayesian Regularization (BR) and Scaled Conjugate Gradient (SCG) training algorithms were also evaluated. The network was trained until the coefficient of correlation (R) was closer to 1.

### Determination of physicochemical properties

#### Alcohol content

The alcohol content of the finished beer was determined using a digital refractometer for brewing (Hanna Instruments (Pty) Ltd., Johannesburg, South Africa). A clean pipette was used to place 0.5–1 ml of the finished beer on the sample well. The Plato readings were recorded afterward.

#### pH

The pH of the finished beverage was determined using a portable pH meter (Hanna Instruments (Pty) Ltd., Johannesburg, South Africa) after calibration with standard buffers of pH 4.00 and 7.00.

#### Total soluble solids

The total soluble solids of the finished beer were determined using a digital refractometer (Hanna Instruments (Pty) Ltd., Johannesburg, South Africa). A clean pipette was used to place 0.5–1 ml of the finished beer on the sample well. The refractive indices of the samples were then recorded accordingly.

#### Viscosity

The consistometer (Endecotts, London, United Kingdom) was used to determine the consistency of the finished beer (cm/min) by pouring 100 ml of the sample into the reservoir behind the gate of the consistometer. The lock release lever was released to instantaneously open the gate, allowing the liquid to flow over the instrument’s graduated scale for 1 min.

#### Total titratable acidity

The American Association of Cereal Chemists (AACC) 02-31^19^ approved method was used to determine the total titratable acidity whereby 10 g of the sample was dissolved in 100 ml distilled water. The solution was well mixed and 0.5 ml of 1% phenolphthalein indicator was added. Finally, standardized 0.1 N sodium hydroxide was used to titrate the prepared solution until a faint pink color was observed. Titratable acidity (in terms of lactic acid %) = volume (ml) required / 20.

### Statistical analysis

All experiments and analyses were conducted in triplicates. ANOVA was employed to determine the significance of the generated models. Design-Expert software version 11.0.0 (Stat-Ease Inc., Minneapolis, USA) was used to determine the Response (Y) of the second-order polynomial equation, the coefficient of determination (R^2^), the ‘predicted R-squared’ and ‘adjusted R-squared’, the coefficient of variance (CV), and the ‘probability F’ value.

### Statement on experimental research and field studies on plants

We confirm that the use of plant-based cereals in our study complied with the relevant institutional, national, and international guidelines and legislation, in particular the IUCN Policy Statement on Research Involving Species at Risk of Extinction.

## Results and discussion

The effect of cooking time, fermentation temperature, and fermentation time on the alcohol content, TSS, TTA, pH, and viscosity were investigated. Optimization of cooking time, fermentation temperature, and fermentation time is essential for maintaining consistent physicochemical properties, curbing undesired changes that may occur during bioprocessing, and understanding the interactions among these process variables at different conditions^1^. In beer production, these are principal factors that influence the final product and its acceptance by consumers^20,21^.

### The effect of input factors on the physicochemical properties of the beer

#### Alcohol content

Samples fermented for a longer period (≥ 60 h) at a relatively higher temperature (≥ 30 °C) contained a lower alcohol content (Table 3, see experimental run numbers 1, 4, 7, 9, 15, and 20). Generally, a higher fermentation temperature affects the rate of sugar metabolism (i.e., leads to a rapid increase in alcohol content and other by-products such as volatile compounds)^21^. On a contrary, in this study, a higher temperature accompanied by a longer fermentation time led to a lower alcohol content (Table 3). Given these conditions, a low alcohol content may be attributed to evaporative ethanol loss. It’s not uncommon for product inhibition to occur during simultaneous saccharification and fermentation, whereby ethanol, a fermentation product, inhibits zymase over time while the products of saccharification inhibit hydrolytic enzymes^22^. In addition, the synthesis of acetate and acids such as formic acid, acetic acid, and levulinic acid at concentrations above 100 mM may inhibit the bioconversion of biomass^22,23^ and thus influence alcohol content.

**Table 3:** Responses from the investigated input parameters.

Exp run	Inputs			Responses				
	Cooking time (hr)	Ferm temp (°C)	Ferm time (hr)	Alcohol (°P)	TSS (g/100 g)	TTA (% lactic acid)	pH	Viscosity (cm/min)
1	2	30	60	8.07^de^ ± 0.70	7.37^cde^ ± 0.31	1.18^hi^ ± 0.03	2.90^ab^ ± 0.05	15.33^cde^ ± 0.58
2	2	30	60	8.70^efg^ ± 0.30	7.73^def^ ± 0.55	1.20^ij^ ± 0.02	2.88^ab^ ± 0.03	14.50^ cd^ ± 0.87
3	3	25	96	7.77^d^ ± 0.75	7.37^cde^ ± 0.60	1.07^ fg^ ± 0.06	2.91^ab^ ± 0.01	10.83^a^ ± 1.04
4	3.68	30	60	6.77^c^ ± 0.06	6.63^c^ ± 0.31	0.81^e^ ± 0.08	2.90^ab^ ± 0.05	17.17^ g^ ± 1.15
5	1	25	96	5.10^a^ ± 0.35	4.90^ab^ ± 0.44	0.81^e^ ± 0.03	2.95^ab^ ± 0.08	19.17^ h^ ± 0.29
6	1	25	24	9.50^hi^ ± 0.20	9.27^ h^ ± 0.31	1.54^ m^ ± 0.04	3.62d^e^ ± 0.02	22.67^j^ ± 0.58
7	0.32	30	60	7.00^c^ ± 0.26	6.70^c^ ± 0.26	0.72^bc^ ± 0.02	2.90^ab^ ± 0.05	25.00^ k^ ± 1.00
8	2	30	0	10.47^j^ ± 0.21	10.3^i^ ± 0.30	0.50^a^ ± 0.03	4.60f. ± 0.20	12.83^b^ ± 0.29
9	2	30	120.54	4.70^a^ ± 0.20	4.80^a^ ± 0.26	0.72^bc^ ± 0.03	3.26^c^ ± 0.02	16.50^ fg^ ± 0.87
10	2	21.59	60	8.73^efg^ ± 0.81	8.37^ fg^ ± 0.65	0.78^cde^ ± 0.05	2.99^b^ ± 0.01	14.17^c^ ± 0.29
11	2	30	60	8.87^fgh^ ± 0.32	7.90^ef^ ± 0.46	1.21^ij^ ± 0.05	2.92^ab^ ± 0.03	16.17^efg^ ± 0.29
12	2	30	60	8.13^de^ ± 0.15	7.87^ef^ ± 0.12	1.13^gh^ ± 0.04	2.91^ab^ ± 0.03	15.67^def^ ± 0.58
13	2	30	60	8.43^ef^ ± 0.29	7.87^ef^ ± 0.38	1.22^ij^ ± 0.02	2.90^ab^ ± 0.07	15.00^ cd^ ± 0.00
14	2	38.41	60	9.50^hi^ ± 0.30	9.60^hi^ ± 0.85	0.79^de^ ± 0.03	2.86^ab^ ± 0.05	17.33^ g^ ± 0.58
15	1	35	96	7.07^c^ ± 0.21	7.03^ cd^ ± 0.15	0.68^b^ ± 0.02	2.81^a^ ± 0.03	14.17^c^ ± 0.29
16	3	35	24	9.27^gh^ ± 0.15	9.03^gh^ ± 0.38	1.35^ k^ ± 0.04	3.36^c^ ± 0.03	14.33^c^ ± 0.58
17	1	35	24	9.17^gh^ ± 0.06	8.93^gh^ ± 0.21	1.44^ l^ ± 0.02	3.73^e^ ± 0.25	20.83^i^ ± 0.76
18	2	30	60	8.37^def^ ± 0.35	8.10^ef^ ± 0.17	1.25^j^ ± 0.01	2.88^ab^ ± 0.03	14.67^ cd^ ± 0.29
19	3	25	24	10.07^ij^ ± 0.12	9.50^ h^ ± 0.78	1.05f. ± 0.05	3.51^d^ ± 0.02	16.83^ g^ ± 0.76
20	3	35	96	5.73^b^ ± 0.15	5.60^b^ ± 0.10	0.74^bcd^ ± 0.02	2.84^ab^ ± 0.04	12.33^b^ ± 0.29

*cm* centimetre, *Exp* experimental, *Ferm* fermentation, *g* gram, *hr* hour, *min* minute, *temp* temperature.

*Each value is a mean of triplicates ± standard deviation of triplicates. Means with no common letters within a row significantly differ (p < 0.05).

#### TSS

Cooking the soured porridge for an adequate amount of time is essential for starch gelatinization and release of locked-up nutrients in yeasts cells^24^. The cooking time was found to influence the alcohol content, TSS, pH, and viscosity (Table 3). The proliferation of fermentative microbes is driven by the hydrolysis of cooked starch to fermentable sugars by endogenous amylolytic enzymes^25^. As the endosperm protein enclosing the starch granules is softened (during gelatinization), moving the grain to the retting water, thereby increasing the amount of TSS^26^. This might explain the increasing trend in the amount of TSS with an increased cooking and fermentation time. As observed from Table 3, cooking for more than 1 h significantly increased the amount of TSS. A reverse trend was observed when the fermentation time was increased. This could be attributed to the growth patterns of microorganisms that correspond to the consumption of soluble solids over time^26^.

The fermentation time largely contributed to the final product’s quality. The longer the fermentation was allowed to proceed, the lower the alcohol content, pH, and viscosity (Table 3). Fermentative microorganisms need sufficient time to adjust to environmental changes for optimal utilization of the substrate for building cellular components (RNA, enzymes, and metabolites)^27^. As cells complete the cell cycle, they enter the exponential growth phase, where they are the healthiest and most uniform, rapidly driving alcoholic fermentation forward^27^. A fermentation time of 24 h was observed to have a relatively higher alcohol content, TSS, and TTA levels compared to 60 h and 96 h. It is possible that within this timeframe fermentative microorganisms entered the exponential phase growth phase which led to a higher microbial activity rate.

#### pH and TTA

The TTA and pH ranged between 0.50–1.54% lactic acid and 2.81–4.60, respectively (Table 3). Generally, *umqombothi* and other African traditional beers have a pH range of 3 to 4.2, and a lactic acid level of 0.26% depending on how the beer is brewed^4,24^. Changes in TTA may be a better measure of the success rate of the fermentation process than changes in pH^26^. A biochemical relationship between alcohol content, TTA, and pH, whereby a lower pH was directly proportional to a high TTA and alcohol content, was observed in this study (Table 3). According to^25^, as the microorganisms carry out alcoholic fermentation, a decrease in the TSS and pH are usually observed. Beers with decreased pH values, such as *umqombothi* (Table 3) have a longer shelf-life, better safety and quality, superior facilitation of microbial growth, and a higher concentration of antimicrobial agents^28^. The low pH and elevated acidity in these beers aid in the elimination of certain pathogenic microorganisms that could pose safety threats^29,30^.

#### Viscosity

Cooking time had a direct influence on the final beer’s viscosity. This is because cooking increasing the availability of starch, which imparts viscidness to food and describes the clarity of the finished beer^31^. In addition, residual starch from incomplete hydrolysis into sugar contributes to a beer’s viscosity^25^. As TTA increases and the pH is lowered, the joint action of malt α-and-β amylases is reduced, thereby reducing the beer’s viscosity, and giving body to the final beer^25^. An increase in the α- amylase, Hitempase 2XL decreased the viscosity in beer produced from malted buckwheat^32^. In western beers, filtration of the beer may be difficult due to high viscosity, thus leading to starch hazes in the final product^32^, while in traditional beers such as *umqombothi*, filtering the beer may result in the loss of important fiber-imparting solids, giving the beer a higher viscosity^4,33,34^.

### Multi-response optimization of process parameters

**Figure 2:**
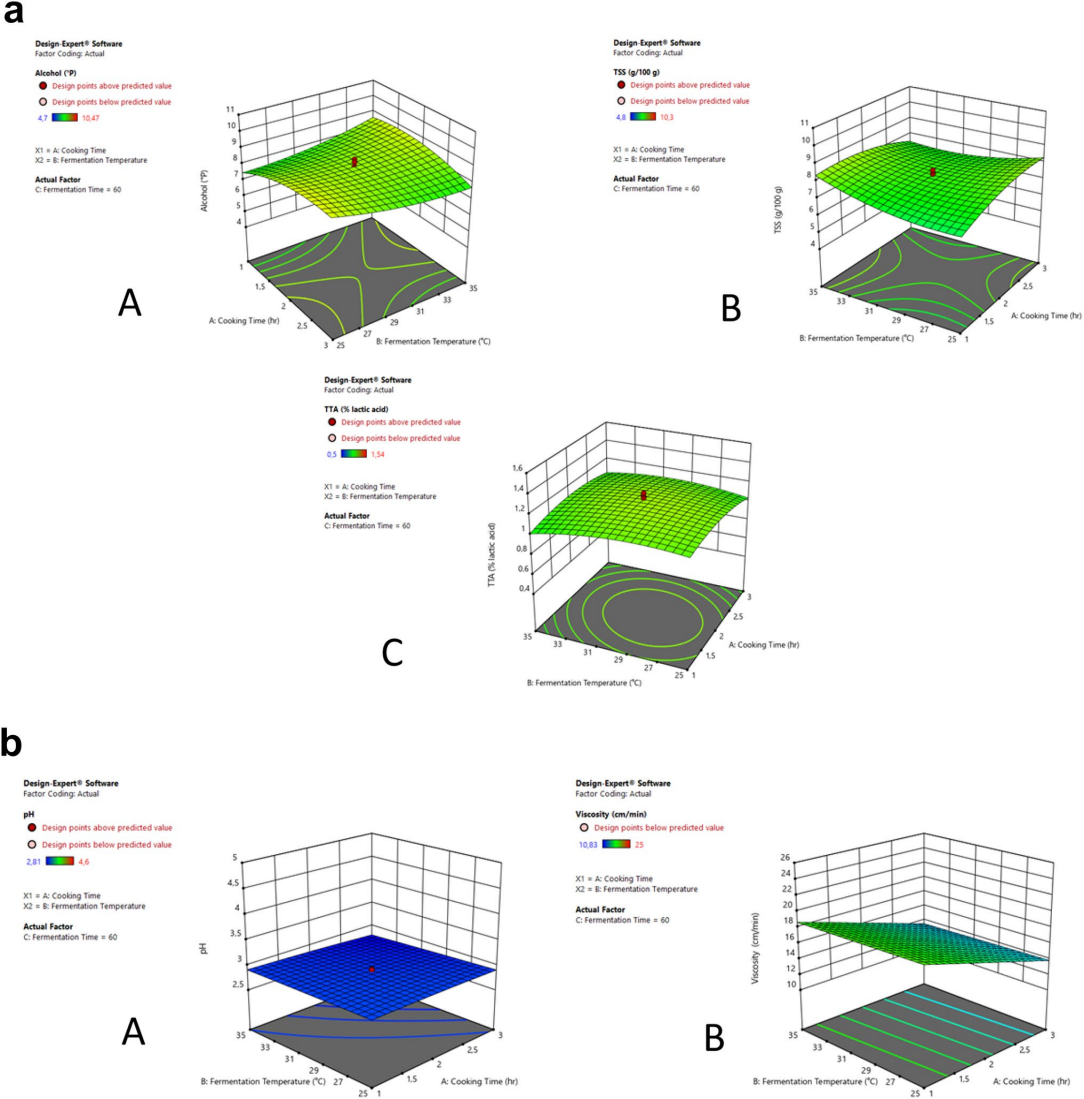
(**a**) 3D response surface plots demonstrating the effect of cooking time, fermentation temperature, and time on *umqombothi* samples: (A) Alcohol content, (B) TSS, (C) TTA. (**b**) A 3D response surface plot demonstrating the effect of cooking time, fermentation temperature, and time on *umqombothi* samples: (A) pH, (B) Viscosity.

In search for the solution, ANOVA, and Fisher’s F-values were used to examine the best fit of the generated RSM models. Model adequacy was determined by the coefficient of determination values (R^2^) and lack of fit tests^1,20^. For the response in view, the R^2^ described the percentage contribution of the process variables (i.e., the amount of variation around the mean explained by the model). For high-confidence prediction purposes, a usable model demands percentage contribution of 88% (R^2^ > 0.88)^35^. The probability of significance was represented by p-values, with a high p-value indicating an inadequate model due to a significant lack of fit^36^. The models for alcohol content, TSS, and pH all had p-values of 0.00, indicating that the lack of fit was insignificant at a 100% confidence level. Polynomial equations together with 3D response surface plots were used to describe the mathematical solutions of the models. Polynomial equations for alcohol content, TSS, TTA, pH, and viscosity are shown in Eqs. (2), (3), (4), (5), and (6), respectively. For better visualization, 3D response surface plots for alcohol content, TSS, TTA, pH, and viscosity are shown in Fig. 2a,b. Regression equations from the fitted models were used to generate 3D plots.

The models for optimizing the alcohol content (°P), TSS (g/100 g) and pH, in the beer, were found to be significant as implied by high model F-values (F ≥ 10) and low p-values (p ≤ 0.05) (Table 4). For the alcohol content and TSS models, X_3_, X_1_X_2_, X_1_^2^ were significant model terms (p ≤ 0.05) (Table 4). Significant model terms for pH were X_3_, X_1_X_3_, X_3_^2^, with p-values of 0.00, 0.047, and 0.00 respectively (Table 4). The predicted determination (pred-R^2^) values for alcohol content and TSS were not as close to the adjusted determination (adj-R^2^) indicating a slight limitation with the model (Table 5). A consideration of outliers, model reduction, and response transformation may improve the empirical model^37^. In contrast, the predicted determination (pred-R^2^) of 0.89 in the pH optimization model was reasonably close to the adjusted determination (adj-R^2^) of 0.97, thus confirming the model’s accuracy in correctly predicting responses (Table 5). Adequate precision values above 4 indicated an adequate signal-to-noise ratio. This means the optimization models for alcohol content, TSS, and pH were suitable to navigate the design space and all of the model’s parameters showed that the developed models were able to predict the responses correctly. The optimization models for alcohol content, TSS had reproducibility above 90% (R^2^ ≥ 0.90) and low coefficient of variation (C.V. %) values (Table 5), indicating a good precision for the capability of the process under evaluation.

**Table 4:** Analysis of variance (ANOVA) for the alcohol content, TSS, TTA, pH, and viscosity quadratic models.

Source	Sum of squares	df	Mean	F-value	p-value
**Alcohol content**					
Model	44.749	9.000	4.972	16.435	0.000*
X_1_–cooking time	0.191	1.000	0.191	0.630	0.446
X_2_–fermentation temperature	0.001	1.000	0.001	0.002	0.964
X_3_–fermentation time	35.501	1.000	35.501	117.348	0.000*
X_1_X_2_	2.509	1.000	2.509	8.293	0.016*
X_1_X_3_	0.054	1.000	0.054	0.180	0.680
X_2_X_3_	0.140	1.000	0.140	0.464	0.511
X_1_^2^	3.870	1.000	3.870	12.791	0.005*
X_2_^2^	1.054	1.000	1.054	3.483	0.092
X_3_^2^	1.028	1.000	1.028	3.399	0.095
Residual	3.025	10.000	0.303		
Lack of fit	2.536	5.000	0.507		
Pure error	0.490	5.000	0.098		
Corrected total sum of squares	47.774	19.000			
**TSS**					
Model	40.620	9.000	4.513	15.685	0.000*
X_1_–cooking time	0.115	1.000	0.115	0.399	0.542
X_2_–fermentation temperature	0.192	1.000	0.192	0.667	0.433
X_3_–fermentation time	32.503	1.000	32.503	112.951	0.000*
X_1_X_2_	2.030	1.000	2.030	7.055	0.024*
X_1_X_3_	0.063	1.000	0.063	0.219	0.650
X_2_X_3_	0.171	1.000	0.171	0.595	0.458
X_1_^2^	2.420	1.000	2.420	8.409	0.016*
X_2_^2^	2.430	1.000	2.430	8.444	0.016*
X_3_^2^	0.126	1.000	0.126	0.437	0.524
Residual	2.878	10.000	0.288		
Lack of fit	2.578	5.000	0.516		
Pure error	0.299	5.000	0.060		
Corrected total sum of squares	43.498	19.000			
**TTA**					
Model	0.678	9.000	0.075	0.819	0.613
X_1_–cooking time	0.001	1.000	0.001	0.009	0.925
X_2_–fermentation temperature	0.004	1.000	0.004	0.047	0.833
X_3_–fermentation time	0.214	1.000	0.214	2.320	0.159
X_1_X_2_	0.005	1.000	0.005	0.054	0.820
X_1_X_3_	0.101	1.000	0.101	1.100	0.319
X_2_X_3_	0.054	1.000	0.054	0.591	0.460
X_1_^2^	0.070	1.000	0.070	0.761	0.404
X_2_^2^	0.057	1.000	0.057	0.614	0.451
X_3_^2^	0.217	1.000	0.217	2.356	0.156
Residual	0.921	10.000	0.092		
Lack of fit	0.912	5.000	0.182		
Pure error	0.008	5.000	0.002		
Corrected total sum of squares	1.599	19.000			
**pH**					
Model	3.710	9.000	0.412	76.174	0.000*
X_1_–cooking time	0.018	1.000	0.018	3.249	0.102
X_2_–fermentation temperature	0.016	1.000	0.016	2.972	0.115
X_3_–fermentation time	1.825	1.000	1.825	337.227	0.000*
X_1_X_2_	0.005	1.000	0.005	0.834	0.383
X_1_X_3_	0.028	1.000	0.028	5.103	0.047*
X_2_X_3_	0.004	1.000	0.004	0.668	0.433
X_1_^2^	0.002	1.000	0.002	0.404	0.539
X_2_^2^	0.000	1.000	0.000	0.032	0.861
X_3_^2^	1.798	1.000	1.798	332.180	0.000*
Residual	0.054	10.000	0.005		
Lack of fit	0.053	5.000	0.011		
Pure error	0.001	5.000	0.000		
Corrected total sum of squares	3.764	19.000			
**Viscosity**					
Model	165.384	9.000	18.376	2.994	0.051
X_1_–cooking time	93.262	1.000	93.262	15.193	0.003*
X_2_–fermentation temperature	0.467	1.000	0.467	0.076	0.788
X_3_–fermentation time	10.541	1.000	10.541	1.717	0.219
X_1_X_2_	4.263	1.000	4.263	0.694	0.424
X_1_X_3_	0.583	1.000	0.583	0.095	0.764
X_2_X_3_	0.088	1.000	0.088	0.014	0.907
X_1_^2^	51.275	1.000	51.275	8.353	0.016*
X_2_^2^	0.000	1.000	0.000	0.000	1.000
X_3_^2^	2.008	1.000	2.008	0.327	0.580
Residual	61.386	10.000	6.139		
Lack of fit	59.399	5.000	11.880		
Pure error	1.986	5.000	0.397		
Corrected total sum of squares	226.770	19.000			

*Significant at p ≤ 0.05.

**Table 5:** Fit statistics of the quadratic model for alcohol content, TSS, TTA, pH, and viscosity optimization.

Parameters	C.V. %	R^2^	Adjusted R^2^	Predicted R^2^	Adequate precision
Alcohol	6.815	0.937	0.880	0.529	13.942
TSS	6.928	0.928	0.874	0.474	13.657
TTA	30.056	0.424	–0.094	–3.335	2.986
pH	2.349	0.986	0.973	0.888	32.559
Viscosity	15.223	0.729	0.486	–1.045	7.005

*C.V* coefficient of variation.

The models for optimizing TTA and viscosity were insignificant as implied by low model F-values (F ≤ 10) and high p-values (p > 0.05) (Fig. 2a,b). Here, model reduction, consideration of outliers, and response transformation will not improve the model. The overall mean may be a better predictor of the designed responses than the current models. A higher-order model may also predict better in certain cases. None of the TTA optimization model terms were significant, while X_1_ and X_1_^2^ were significant model terms (p ≤ 0.05) for viscosity. Both the models’ limitations were described by significant differences between the predicted determination. The TTA model had a pred-R^2^ of –3.34 and an adj-R^2^ of –0.09. Similarly, the model for viscosity had a pred-R^2^ of –1.05 and an adj-R^2^ of 0.49. In this case, a negative predicted determinant (pred-R^2^) implies that the overall mean may be a better predictor of the designed response than the current model^38^. A higher-order model may also predict better in certain cases. An adequate precision value of 2.99 in the TTA model indicated an undesirable signal-to-noise ratio. This means the model was not suitable to navigate the design space. The viscosity optimization model had an adequate precision above 4, meaning the model was suitable for navigating the design space. The low reproducibility of 42% (Table 5) for the TTA optimization model was indicated by a low coefficient of determination (R^2^ = 0.424). In contrast, the coefficient of determination for the viscosity was 0.729, representing a 73% reproducibility. Although the reproducibility can be considered adequate, a C.V. % value of 15.22 may be alarming (Table 5). From the obtained experimental data, second-order polynomial equations showing the significance of linear, quadratic, and interactive terms in predicting the response were generated and shown in Eqs. (2), (3), (4), (5), and (6) below:

2 $${\text{Y}}_{1} = 8.4244 + 0.118123{\text{X}}_{1} + 0.00695478{\text{X}}_{2} { }{-}1.61535{\text{X}}_{3} { }{-}0.56{\text{X}}_{1} {\text{X}}_{2} + 0.0825{\text{X}}_{1} {\text{X}}_{3} + { }0.1325{\text{X}}_{2} {\text{X}}_{3} { }{-}0.518085{\text{X}}_{1}^{2} + 0.270339{\text{X}}_{2}^{2} { }{-}0.269005{\text{X}}_{3}^{2}$$

3 $${\text{Y}}_{2} = 7.80809 + 0.0916957{\text{X}}_{1} + 0.11852{\text{X}}_{2} { }{-}1.54563{\text{X}}_{3} { }{-}0.50375{\text{X}}_{1} {\text{X}}_{2} + 0.08875{\text{X}}_{1} {\text{X}}_{3} + 0.14625{\text{X}}_{2} {\text{X}}_{3} { }{-}0.4097{\text{X}}_{1}^{2} + 0.410543{\text{X}}_{2}^{2} { }{-}0.0940759{\text{X}}_{3}^{2}$$

4 $${\text{Y}}_{3} = 1.18401{ }{-}0.00795488{\text{X}}_{1} { }{-}0.0178066{\text{X}}_{2} { }{-}0.125284{\text{X}}_{3} + 0.025{\text{X}}_{1} {\text{X}}_{2} + 0.1125{\text{X}}_{1} {\text{X}}_{3} { }{-}0.0825{\text{X}}_{2} {\text{X}}_{3} { }{-}0.0697051{\text{X}}_{1}^{2} { }{-}0.0626341{\text{X}}_{2}^{2} { }{-}0.123537{\text{X}}_{3}^{2}$$

5 $${\text{Y}}_{4} = 2.90059{ }{-}0.0358794{\text{X}}_{1} { }{-}0.0343149{\text{X}}_{2} { }{-}0.36624{\text{X}}_{3} { }{-}0.02375{\text{X}}_{1} {\text{X}}_{2} + 0.05875{\text{X}}_{1} {\text{X}}_{3} { }{-}0.02125{\text{X}}_{2} {\text{X}}_{3} { }{-}0.0123214{\text{X}}_{1}^{2} { }{-}0.00348255{\text{X}}_{2}^{2} + 0.355683{\text{X}}_{3}^{2}$$

6 $${\text{Y}}_{5} = 15.2439{ }{-}2.61323{\text{X}}_{1} { }{-}0.184928{\text{X}}_{2} { }{-}0.880224{\text{X}}_{3} + 0.73{\text{X}}_{1} {\text{X}}_{2} + 0.27{\text{X}}_{1} {\text{X}}_{3} + 0.105{\text{X}}_{2} {\text{X}}_{3} + 1.8859{\text{X}}_{1}^{2} { }{-}0.000308033{\text{X}}_{2}^{2} { }{-}0.375946{\text{X}}_{3}^{2}$$

where Y_1_ = response for alcohol content (°P), Y_2_ = response for TSS (g/100 g), Y_3_ = response for TTA (% lactic acid), Y_4_ = response for pH, Y_5_ = response for viscosity (cm/min), X_1_ = Cooking time (hr), X_2_ = Fermentation temperature (°C), X_3_ = Fermentation time (hr).

### The effect of input factors on the physicochemical properties of the optimal beer brew

Independent variables, cooking time (hr) coded as (X_1_), fermentation temperature (°C) coded as (X_2_), and time (hr) coded as (X_3_) were optimized. The optimization goal for all independent variables was set to ‘target’ as dictated by the nature of the study. The responses alcohol content (°P), TSS (g/100 g), TTA (% lactic acid), pH, and viscosity (cm/min) were considered for optimization. The software generated 100 optimization solutions each with a desirability value of 1. To select a suitable solution, prediction values of each solution were compared to prediction values of the constructed ANN. Yeast survival and proliferation, under-and-over cooking, shelf-life associated spoilage, and conditions’ applicability in real-life (study objectives) were also considered. Taking these variables into account, a solution that favored these considerations was selected. A cooking time of 1.1 h, fermentation temperature of 29.3 °C, and fermentation time of 25.9 h were optimal bioprocessing conditions. The parameters (alcohol content, TSS, TTA, pH, and viscosity) were subsequently investigated and the results are provided in Table 6. The customary brew (CB) was prepared by cooking the mixed ingredients for 30 min and leaving the cooked slurry to ferment at 25 °C for 24 h. The CB was then compared with the optimized brew (OPB).

**Table 6:** Physicochemical properties of *umqombothi*.

Sample	Alcohol (°P)	TSS (g/100 g)	TTA (% lactic acid)	pH	Viscosity (cm/min)
CB	11.33 ± 0.21^a^	10.90 ± 0.10^a^	0.57 ± 0.02^a^	4.23 ± 0.02^b^	16.83 ± 0.76^b^
OPB	13.63 ± 0.12^b^	13.33 ± 0.21^b^	0.68 ± 0.02^b^	3.27 ± 0.03^a^	11.50 ± 0.87^a^

*CB* customary brew, *OPB* optimized brew. Each value is a mean ± standard deviation of triplicates.

*Each value is a mean of triplicates ± standard deviation of triplicates. Means with no common letters within a row significantly differ (p < 0.05).

The OPB was found to have a low pH (3.27 ± 0.03) compared to the CB (4.23 ± 0.02) (Table 6). As a result, the OPB had a higher alcohol content (13.63 ± 0.12°P) and a higher TTA (0.68 ± 0.02% lactic acid). In preparing high-quality *umqombothi*, a 60 min cooking time has been suggested to be ideal^39^. A cooking time of 1.1 h did not under-/over-gelatinize the starch and provided adequate nutrients to yeasts cells^24^. In addition, the achieved gelatinization improved water absorption into the granules, thereby improving the viscosity^40^. This was reflected in the viscosity obtained for the OPB, which had more a desirable viscosity value compared to the CB (Table 6). A fermentation temperature of 29.3 °C was optimal for higher production of alcohol in the OPB (Table 6). A higher TSS in the OPB (Table 6) described the type of sugar conversion and its dependence on temperature for a rich, finished beer^41^. The slightly higher fermentation temperature and a relatively short fermentation time in the OPB appeared to improve the overall physicochemical properties of *umqombothi*. A fermentation time of 25.9 h was ideal for the fermentation rate and final beer.

### ANN training, validation, and testing on experimental responses

An appropriate ANN construction involves the selection of network architecture, determination of hidden layers and number of neurons in each layer, learning—training—validation, and verification of the data^18^. In building a better ANN model, the number of the hidden layers between inputs and output must be appropriately trained and fitted^18^. To achieve this, the number of neurons in the hidden was varied (i.e., 5, 10, and 20 neurons in the hidden layer) (data not reported). To further study the responses of the model, three different training algorithms were evaluated. When 10 neurons in the hidden layer were used, all the algorithms rapidly generated solutions with high R and R^2^ values (data not reported). However, when the neurons were increased to 20, the number of reiterations increased in the BR algorithm, thus taking longer to generate a solution. In contrast, both the LM and SCG algorithms were not significantly affected by an increase or decrease in the number of neurons and maintained a higher rapidity in generating solutions. The SCG uses second-order approximation, resulting in fewer iterations and faster learning^42^. This may be due to the algorithm using a step-size scaling mechanism that avoids a timewasting line search per learning iteration^43,44^.

Adequate training, validation, testing, and overall prediction accuracy were observed when the LM algorithm was used (Table 7). The LM algorithm which may be the fastest of the three training algorithms specifically works with loss functions presented in the form of a sum of squared errors (SSE)^45,46^. Unfortunately, LM cannot be applied to the cross-entropy error and the root mean squared error functions^46^. For functioning approximation problems, the LM training algorithm was able to obtain lower MSE than all other algorithms among regularization techniques. As a result, the LM is the recommended choice with better performance in terms of rapidity and the overfitting problem when there are a few thousand instances and a few hundred parameters for training the ANN^46,47^. In an unrelated study, the LM training algorithm was found to show the highest accuracy in comparison to different training algorithms in a MLP model that forecasted chemical elements distribution in the topsoil^45^.

**Table 7:** Training, validation, and testing performance indices.

	Alcohol			TSS			TTA			pH			Viscosity		
	MSE	R	R^2^	MSE	R	R^2^	MSE	R	R^2^	MSE	R	R^2^	MSE	R	R^2^
Training	0.01	1.00	1.00	0.01	1.00	0.99	0.00	0.98	0.96	0.35	1.00	1.00	0.21	0.99	0.99
Validation	0.42	0.91	0.83	0.09	0.98	0.96	0.01	0.95	0.91	0.02	1.00	1.00	0.50	0.97	0.94
Testing	0.33	0.77	0.60	0.45	0.97	0.93	0.08	0.72	0.52	1.44	0.53	0.28	4.97	0.87	0.76
Overall	0.42	0.98	0.96	0.09	0.98	0.96	0.01	0.90	0.81	0.02	0.71	0.50	0.50	0.96	0.92

*MSE* mean squared error, *R* coefficient of correlation, *R*^*2*^ coefficient of determination, *TSS* total soluble solids, *TTA* total titratable acidity.

**Figure 3:**
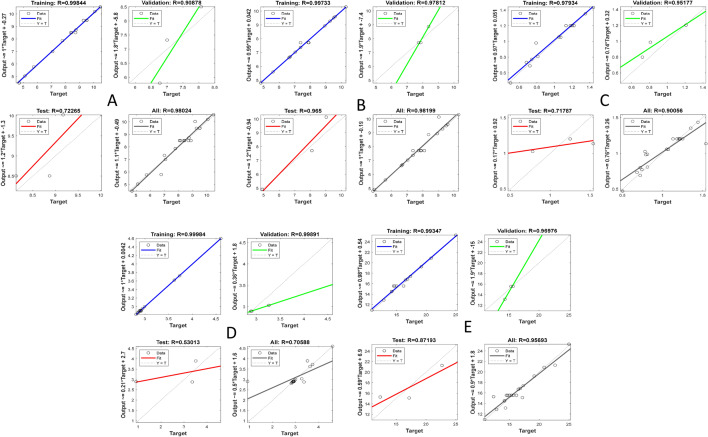
Response plots of the ANN: (**A**) Alcohol content (°P), (**B**) TSS (g/100 g), (**C**) TTA (% lactic acid), (**D**) pH, (**E**) viscosity (cm/min).

The ANN training using the LM algorithm stopped automatically when generalization stopped, indicated by an increase in the MSE of the validation samples. In measuring performance indices of the ANN, the MSE is the most used and simplest error function^48,49^. The MSE measures the ability of the model to predict responses accurately, with a lower MSE showing a higher modeling ability^18^. In combination, R^2^ and MSE evaluated the overall accuracy of the model^18^. The coefficient of correlation (R) was used to measure the correlation between inputs and targets. R = 1 described a close relationship, and R = 0 described a random relationship. ANN models for alcohol content, TSS, TTA, and viscosity had overall R^2^ values of 0.96, 0.96, 0.81, and 0.92, respectively (Table 7). These values were closer to 1, suggesting high reliability in model prediction accuracy. The overall R^2^ value for pH was 0.50 representing a 50% reproducibility. Overall, a high correlation between inputs and targets was observed for alcohol content (0.98), TSS (0.98), TTA (0.90), and viscosity (0.96) (Fig. 3).

**Figure 4:**
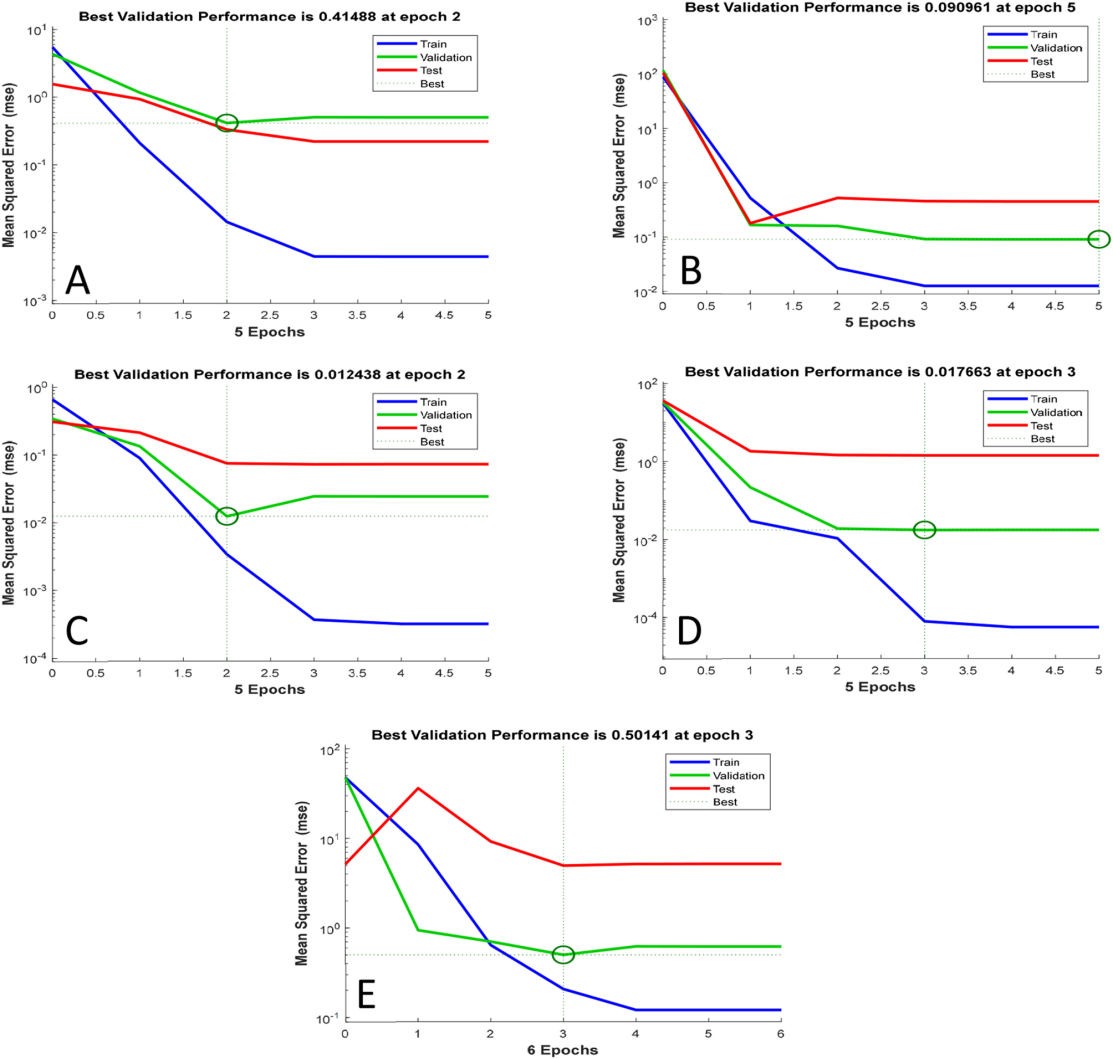
Validation performance plot of the ANN: (**A**) Alcohol content (°P), (**B**) TSS (g/100 g), (**C**) TTA (% lactic acid), (**D**) pH, (**E**) viscosity (cm/min).

Apart from MSE values, the ANN was further assessed using performance curves. Performance curves display the network’s incremental training process and the direction in which it learns. These curves plot training record error values against the number of training epochs. Consequently, the learning curve is a plot describing a model learning performance over time or experience^50^. Performance curves are useful in diagnosing problems with learning aspects such as unrepresentative training datasets, underfitting models, unrepresentative validation datasets, and overfitting models^50^. The ANN best validation performance curves for the responses are shown in Fig. 4**.** The ANN achieved the best learning and the lowest error after a few iterations (epochs). The best validation performance for each network was taken from the epoch with the lowest validation error. Both alcohol and TTA had the shortest iterations before achieving the best validation performance. In contrast, TSS achieved its best validation performance at epoch 5. After more epochs of training, the error is generally reduced but may start to increase on the validation dataset as overfitting of the training data occurs^51^. All the networks showed a good learning rate for the training stage and a high learning rate for the validation and testing stages^52^. In addition, both the training and validation showed a good fit displayed by training and validation MSE (loss) values which decreased to a point of stability with relatively nominal gaps between the two final MSE (or loss) values^50^. Overall better learning is described by error scores closer to 0, thus indicating that the training dataset was learned thoroughly and minimal mistakes were made^50^.

### Comparison between the RSM and ANN responses

An optimization prediction model developed by RSM was assessed by comparing its prediction accuracy with that of the ANN which was also used to validate the entire process. Table 8 shows the error comparison obtained from both and ANN predictions. The comparative error analysis was used to verify the prediction accuracy and generalization capacity of both models in optimizing the bioprocess^53,54^. Overall, the ANN model showed lower error values than the RSM, indicating lower computational deviations and an advanced generalization capability^11,54^. As a result, ANN displayed a higher prediction accuracy and better model fitting^18^. On the other hand, RSM prediction values can be accepted with a higher degree of confidence since they are closer to experimental values and ANN prediction values^18,55^. The results from Table 8 show a close correlation between the experimental values and RSM and ANN’s predicted values. Both RSM and ANN models showed a relatively high number of inexact predictions for viscosity.

**Table 8:** RSM and ANN predictions values.

Run	Alcohol (°P)					TSS (g/ 100 g)					TTA (% lactic acid)					`pH					Viscosity (cm/min)				
	Exp	RSM Pred	Error RSM	ANN Pred	Error ANN	Exp	RSM Pred	Error RSM	ANN Pred	Error ANN	Exp	RSM Pred	Error RSM	ANN Pred	Error ANN	Exp	RSM Pred	Error RSM	ANN Pred	Error ANN	Exp	RSM Pred	Error RSM	ANN Pred	Error ANN
1	8.07	8.738	0.243	10.57	− 0.10	7.37	8.117	0.237	10.30	0.00	1.18	1.115	0.134	0.47	0.03	2.90	2.914	0.032	4.60	0.00	15.33	14.138	1.094	12.85	− 0.02
2	8.70	8.424	0.224	8.72	0.01	7.73	7.808	0.219	8.87	− 0.50	1.20	1.184	0.124	1.02	− 0.24	2.88	2.901	0.030	3.00	− 0.01	14.50	15.244	1.010	14.50	− 0.33
3	7.77	8.873	0.450	7.00	0.07	7.37	8.732	0.439	7.03	0.00	1.07	1.023	0.248	0.73	− 0.05	2.91	3.475	0.060	2.81	0.00	10.83	15.191	2.029	14.47	− 0.30
5	6.77	6.072	0.450	7.86	− 0.09	6.63	6.111	0.439	7.37	0.00	0.81	0.832	0.248	1.05	0.02	2.90	2.818	0.060	2.91	0.00	17.17	14.180	2.028	10.93	− 0.10
4	5.10	5.392	0.450	5.79	0.98	4.90	5.220	0.439	6.63	0.00	0.81	0.824	0.248	0.81	0.00	2.95	2.883	0.060	2.90	0.00	19.17	19.026	2.028	15.11	2.06
6	9.50	6.913	0.450	9.49	0.01	9.27	6.589	0.439	9.27	0.00	1.54	0.983	0.248	1.13	0.41	3.62	2.976	0.060	3.62	0.00	22.67	12.880	2.028	21.27	1.40
7	7.00	10.244	0.450	8.50	− 0.43	6.70	9.795	0.439	7.71	− 0.34	0.72	0.843	0.248	1.20	− 0.02	2.90	3.549	0.060	2.90	0.00	25.00	14.310	2.029	15.55	− 0.22
8	10.47	9.922	0.450	5.77	− 0.04	10.30	9.734	0.439	5.60	0.00	0.50	1.214	0.248	0.77	− 0.03	4.60	3.712	0.060	2.85	− 0.01	12.83	19.497	2.029	15.28	− 2.95
9	4.70	6.791	0.450	5.03	0.07	4.80	6.757	0.439	4.92	− 0.02	0.72	0.573	0.248	0.99	− 0.18	3.26	2.820	0.060	2.95	0.00	16.50	17.407	2.028	19.22	− 0.05
10	8.73	8.704	0.233	8.50	0.20	8.37	8.287	0.228	7.71	0.02	0.78	1.146	0.129	1.20	0.00	2.99	2.922	0.031	2.90	− 0.02	14.17	14.761	1.051	15.55	− 1.05
11	8.87	8.440	0.221	10.02	− 0.85	7.90	7.927	0.215	8.93	0.00	1.21	1.157	0.122	1.43	0.01	2.92	2.881	0.030	3.72	0.01	16.17	14.615	0.995	20.86	− 0.03
12	8.13	9.333	0.264	8.50	0.37	7.87	8.953	0.258	7.71	0.19	1.13	1.170	0.146	1.20	0.01	2.91	3.275	0.035	2.90	0.02	15.67	14.926	1.190	15.55	0.62
13	8.43	9.448	0.241	7.32	− 0.32	7.87	8.870	0.235	6.70	0.00	1.22	1.113	0.133	0.80	− 0.08	2.90	3.324	0.032	2.89	0.01	15.00	14.645	1.084	25.28	− 0.28
14	9.50	9.729	0.250	8.50	− 0.37	9.60	9.178	0.244	7.71	0.16	0.79	1.158	0.138	1.20	− 0.07	2.86	3.489	0.033	2.90	− 1.99	17.33	16.216	1.125	15.55	0.12
15	7.07	6.685	0.257	8.50	− 0.07	7.03	6.318	0.251	7.71	0.16	0.68	0.999	0.142	1.20	0.02	2.81	2.848	0.034	2.90	0.00	14.17	13.487	1.157	15.55	− 0.55
16	9.27	9.064	0.220	9.48	0.02	9.03	8.488	0.215	9.60	0.00	1.35	1.202	0.121	0.98	− 0.19	3.36	3.116	0.029	2.86	0.00	14.33	15.047	0.992	17.39	− 0.06
17	9.17	6.870	0.235	4.48	0.22	8.93	6.462	0.229	4.80	0.00	1.44	1.002	0.129	0.69	0.03	3.73	2.840	0.031	3.03	0.23	20.83	13.651	1.057	16.70	− 0.20
18	8.37	9.466	0.237	10.19	− 0.12	8.10	8.956	0.231	9.50	0.00	1.25	1.238	0.131	1.06	− 0.01	2.88	3.482	0.032	3.89	− 0.38	14.67	17.316	1.069	16.86	− 0.03
19	10.07	7.076	0.236	8.50	− 0.13	9.50	6.612	0.230	7.71	0.39	1.05	1.054	0.130	1.20	0.05	3.51	2.825	0.032	2.90	− 0.02	16.83	13.623	1.061	15.55	− 0.88
20	5.73	8.813	0.240	9.48	− 0.21	5.60	8.314	0.235	10.13	− 1.10	0.74	1.134	0.133	1.35	0.00	2.84	3.113	0.032	2.88	0.48	12.33	14.704	1.083	13.13	1.20

*cm* centimetre, *Exp* experimental, *g* gram, *hr* hour, *min* minute, *ml* millimetre, *Pred* predicted, *TSS* total soluble solids, *TTA* total titratable acidity.

The difference between predicted and experimental values directly contributed to the extent deviation in predictive capacity of each model. While RSM is recommended for modelling new processes, its sensitivity may be limited^55^. Despite this limitation, RSM has an obvious way of showing the effect of individual elements and their interactions on a specific system^11^. For example, the effect on a specific parameter is shown by a greater higher value of coefficients in ANOVA^57^. On the other hand, a higher number of inputs are required for ANN than RSM to have better predictions^55^. ANN cannot give such insights into the system directly since it is a ‘black box’^56^. Nonetheless, ANN can universally describe high-level interactions in non-linear systems without prior specification for suitable fitting function^55,57^. Additionally, ANN can calculate multi-responses in a single process^53^. As depicted by the close agreement between the experimental and predicted values, RSM and ANN are adequate for developing a bioprocess that optimally produces *umqombothi*. Advanced soft computing approaches like ANN may be preferred in the case of data sets with a limited number of observations in which regression models fail to capture reliably^18^. The closeness of the experimental values and predicted suggest that the non-linear fitting effects of the model are good, recommending the use of the proposed procedure^18,57^. A coupled modeling approach can thus be applied in bioprocess method development for *umqombothi* and related variations.

## Conclusion

Both RSM and ANN were effective bioprocess development tools that facilitated the optimization of *umqombothi*. The effectiveness of RSM was shown by R^2^ closer to 1. The R^2^ values were 0.94, 0.93, 0.99, and 0.73 for alcohol, TSS, pH, and viscosity respectively, showing reliability and reproducibility above 70%. Similarly, ANN displayed a high degree of accuracy. Constructed ANN models for alcohol, TSS, TTA, and viscosity had R^2^ values of 0.96, 0.96, 0.81, and 0.92 respectively. As result, a good correlation between the experimental and predicted values suggests that a coupled approach may positively impact the bioprocess and the final product. However, further investigation of other key parameters (i.e., starter culture, the content and ratio of raw materials, souring time and temperature, and cooking temperature) is still required. Furthermore, the use of an additional tool such as genetic algorithm may resolve computational and modeling limitations.

## Data Availability

The datasets generated during and/or analyzed during the current study are available from the corresponding author on reasonable request.

## References

[1] Nwabueze TU (2010). Basic steps in adapting response surface methodology as mathematical modelling for bioprocess optimization in the food systems. Int. J. Food Sci..

[2] Madamba PS (2002). The response surface methodology: an application to optimize dehydration operations of selected agricultural crops. LWT..

[3] Takahashi MB, de Coelho OH, Fernández Núñez EG, Rocha JC (2019). Brewing process optimization by artificial neural network and evolutionary algorithm approach. J. Food Process Eng..

[4] Hlangwani E, Adebiyi JA, Doorsamy W, Adebo OA (2020). Processing, characteristics and composition of umqombothi (a South African traditional beer). Processes..

[5] De Filippis LAC, Serio LM, Facchini F, Mummolo G (2017). ANN modelling to optimize manufacturing process. Advanced Applications for Artificial Neural Networks.

[6] Kana EBG, Oloke JK, Lateef A, Oyebanji A (2012). Comparative evaluation of artificial neural network coupled genetic algorithm and response surface methodology for modeling and optimization of citric acid production by *Aspergillus niger* MCBN297. Chem. Eng. Trans..

[7] Sewsynker-Sukai Y, Faloye F, Kana EBG (2017). Artificial neural networks: an efficient tool for modelling and optimization of biofuel production (a mini review). Biotechnol. Biotechnol. Equip..

[8] Adinarayana K, Ellaiah P, Srinivasulu B, Devi RB, Adinarayana G (2003). Response surface methodological approach to optimize the nutritional parameters for neomycin production by *Streptomyces marinensis* under solid-state fermentation. Process Biochem..

[9] Behera SK, Meena H, Chakraborty S, Meikap BC (2018). Application of response surface methodology (RSM) for optimization of leaching parameters for ash reduction from low-grade coal. Int. J. Min. Sci. Technol..

[10] Mewa-Ngongang M, du Plessis HW, Hutchinson UF, Mekuto L, Ntwampe SK (2017). Kinetic modelling and optimization of antimicrobial compound production by *Candida pyralidae* KU736785 for control of *Candida guilliermondii*. Food Sci. Technol. Int..

[11] Youssefi S, Emam-Djomeh Z, Mousavi SM (2009). Comparison of artificial neural network (ANN) and response surface methodology (RSM) in the prediction of quality parameters of spray-dried pomegranate juice. Dry. Technol..

[12] Basheer IA, Hajmeer M (2000). Artificial neural networks: fundamentals, computing, design, and application. J. Microbiol. Methods..

[13] Kukreja H, Bharath N, Siddesh CS, Kuldeep S (2016). An introduction to artificial neural network. Int. J. Adv. Res. Innov. Ideas Educ..

[14] Littmann M (2020). Validity of machine learning in biology and medicine increased through collaborations across fields of expertise. Nat. Mach. Intell..

[15] Khadir MT (2021). Artificial neural networks for food processes: a survey. De Gruyter.

[16] Reyed R (2019). Computational biotechnology: An approach in silico based modeling bioprocess. IJRSMB..

[17] Yolmeh M, Jafari SM (2017). Applications of response surface methodology in the food industry processes. Food Bioproc. Tech..

[18] Ram Talib NS, Halmi MIE, Abd Ghani SS, Zaidan UH, Shukor MYA (2019). Artificial neural networks (ANNs) and response surface methodology (RSM) approach for modelling the optimization of chromium (VI) reduction by newly isolated *Acinetobacter radioresistens* strain NS-MIE from agricultural soil. Biomed Res. Int..

[19] AACC International (2010). Approved Methods of Analysis: Method 02-31.01.

[20] Puerari C, Strejc J, Souza AC, Karabín M, Schwan RF, Brányik T (2016). Optimization of alcohol-free beer production by lager and cachaça yeast strains using response surface methodology. J. Inst. Brew..

[21] Kucharczyk K, Tuszyński T (2018). The effect of temperature on fermentation and beer volatiles at an industrial scale. J. Inst. Brew..

[22] Amadi PU, Ifeanacho MO (2016). Impact of changes in fermentation time, volume of yeast, and mass of plantain pseudo-stem substrate on the simultaneous saccharification and fermentation potentials of African land snail digestive juice and yeast. J. Genet. Eng. Biotechnol..

[23] Larsson S (1999). The generation of fermentation inhibitors during dilute acid hydrolysis of softwood. Enzyme Microb. Technol..

[24] Nanadoum M, Pourquie J, Preedy V (2009). Sorghum Beer: Production, Nutritional Value, and Impact upon Human Health. Beer in Health and Disease Prevention.

[25] Muyanja C, Namugumya BS (2009). Traditional processing, microbiological, physicochemical, and sensory characteristics of kwete, a Ugandan fermented maize-based beverage. Afr. J. Food Agric. Nutr..

[26] Alphonce S, Kaale LD (2020). Assessment of biochemical changes during fermentation process for production of traditional fermented cassava meal “Mchuchume”. Tanz. J. Sci..

[27] Bruslind, L. Microbial Growth https://bio.libretexts.org/Bookshelves/Microbiology/Book%3A_Microbiology_(Bruslind)/09%3A_Microbial_Growth (2021).

[28] Adekoya I, Obadina A, Adaku CC, De Boevre M, Okoth S, De Saeger S, Njobeh P (2018). Mycobiota and co-occurrence of mycotoxins in South African maize-based opaque beer. Int. J. Food Microbiol..

[29] Abdoul-Latif FM, Bassolé IH, Dicko MH (2013). Proximate composition of traditional local sorghum beer “dolo” manufactured in Ouagadougou. Afr. J. Biotechnol..

[30] Adebiyi JA, Kayitesi E, Adebo OA, Changwa R, Njobeh PB (2019). Food fermentation and mycotoxin detoxification: An African perspective. Food Control.

[31] Abd Elmoneim OE, Bernhardt R, Cardone G, Marti A, Iametti S, Marengo M (2017). Physicochemical properties of sorghum flour are selectively modified by combined germination-fermentation. J. Food Sci. Technol..

[32] Phiarais BPN, Schehl BD, Oliveira JC, Arendt EK (2006). Use of response surface methodology to investigate the effectiveness of commercial enzymes on buckwheat malt for brewing purposes. J. Inst. Brew..

[33] Ikalafeng, B.K. Microbiota and mycotoxins in traditional beer of the greater Kimberley area and associated brewing and consumption practices. Doctoral dissertation. Bloemfontein: Central University of Technology (2008).

[34] Lues JFR, Ikalafeng B, Maharasoa M, Shale K, Pool E (2009). Brewing and consumptions practices of indigenous traditional beer in a typical South African semi-urban area. IAJIKS..

[35] Adebo OA, Njobeh PB, Mulaba-Bafubiandi AF, Adebiyi JA, Desobgo ZSC, Kayitesi E (2018). Optimization of fermentation conditions for ting production using response surface methodology. J. Food Process. Preserv..

[36] Kumar S, Kohli D, Joshi J, Wilson I (2019). Response surface optimization of fermenting parameters for the production of beer from finger millet and apple juice by using Box-Behnken Design. Carpath. J. Food Sci. Technol..

[37] Rheem S, Oh S (2019). Improving the quality of response surface analysis of an experiment for coffee-supplemented milk beverage: I. Data screening at the center point and maximum possible R-square. Food Sci. Anim. Resour..

[38] Statease.com. General Sequence of Analysis: Fit Summary (RSM/MIX Model Selection) https://www.statease.com/docs/v11/contents/analysis/fitsummary/#:~:text=The%20F%2Dvalue%20compares%20the,design%20points%20(Pure%20Error) (2021).

[39] Sitole D (2021). Dorah Sitole: 40 Years of Iconic Food.

[40] Santos, D. Starch Gelatinization. Science Meets Food https://sciencemeetsfood.org/starch-gelatinization/ (2013)

[41] Aroh K (2018). Review: Beer production. SSRN Electron. J..

[42] Babani L, Jadhav S, Chaudhari B (2016). Scaled conjugate gradient-based adaptive ANN control for SVM-DTC induction motor drive. In IFIP Int. Conf. Artif. Intell. Appl. Innov..

[43] Møller MF (1993). A scaled conjugate gradient algorithm for fast supervised learning. Neural Netw..

[44] Fatemi M (2017). A scaled conjugate gradient method for nonlinear unconstrained optimization. Optim Methods Softw..

[45] Shichkin A (2018). Training algorithms for artificial neural network in predicting of the content of chemical elements in the upper soil layer. In AIP Conf. Proc..

[46] Quesada, A. 5 Algorithms to Train a Neural Network https://www.neuraldesigner.com/5_algorithms_to_train_a_neural_network (2015)

[47] Kayri M (2016). Predictive abilities of Bayesian regularization and Levenberg-Marquardt algorithms in artificial neural networks: a comparative empirical study on social data. MCA..

[48] Christiansen NH, Voie PET, Winther O, Høgsberg J (2014). Comparison of neural network error measures for simulation of slender marine structures. J. Appl. Math..

[49] Grover, P. 5 Regression Loss Functions All Machine Learners Should Know https://heartbeat.fritz.ai/5-regression-loss-functions-all-machine-learners-should-know4fb140e9d4b0 (2018)

[50] Brownlee, J. How to use Learning Curves to Diagnose Machine Learning Model Performance https://machinelearningmastery.com/learning-curves-for-diagnosing-machine-learning-model-performance/ (2019)

[51] MathWorks. Plot network performance - MATLAB plotperform https://www.mathworks.com/help/deeplearning/ref/plotperform.html (2021).

[52] Karpathy, A. CS23 1n Convolutional Neural Networks for Visual Recognition https://cs231n.github.io/neural-networks-3/#loss (2021).

[53] Desai KM, Survase SA, Saudagar PS, Lele SS, Singhal RS (2008). Comparison of artificial neural network (ANN) and response surface methodology (RSM) in fermentation media optimization: A case study of fermentative production of scleroglucan. Biochem. Eng. J..

[54] Nazerian M, Kamyabb M, Shamsianb M, Dahmardehb M, Kooshaa M (2018). Comparison of response surface methodology (RSM) and artificial neural networks (ANN) towards efficient optimization of flexural properties of gypsum- bonded fiberboards. Cerne.

[55] Shafi J, Sun Z, Ji M, Gu Z, Ahmad W (2018). ANN and RSM based modelling for optimization of cell dry mass of Bacillus sp strain B67 and its antifungal activity against Botrytis cinerea. Biotechnol. Biotechnol. Equip..

[56] Jawale K, Bose PSC, Rao CSP (2015). Use of ANN and RSM to model, predict and optimize the performance parameters for turning waspaloy. IJAERS..

[57] Patel KA, Brahmbhatt PK (2016). A comparative study of the RSM and ANN models for predicting surface roughness in roller burnishing. Proc. Technol..

